# Effect of conjugated linoleic acid and vitamin E on glycemic control, body composition, and inflammatory markers in overweight type2 diabetics

**DOI:** 10.1186/2251-6581-12-42

**Published:** 2013-07-20

**Authors:** Zhaleh Shadman, Forough Azam Taleban, Navid Saadat, Mehdi Hedayati

**Affiliations:** 1 National Nutrition and Food Technology Research Institute (NNFTRI), Department of Human Nutrition, Faculty of Nutrition Sciences and Food Technology, Shahid Beheshti University of Medical Sciences, Tehran, Iran; 2 Endocrine Research Center, Research Institute for Endocrine Sciences, Shahid Beheshti University of Medical Sciences, Tehran, Iran; 3 Cellular and Molecular Endocrine Research Center, Research Institute for Endocrine Sciences, Shahid Beheshti University of Medical Sciences, Tehran, Iran

**Keywords:** CLA, VitE, Inflammatory factors, Type2 diabetes

## Abstract

**Background:**

The healthy properties of conjugated linoleic acid (CLA) such as weight loss, reducing cardiovascular risk factors and inflammation have been reported. The trans-10, cis-12 CLA isomer is related to increasing insulin resistance, but the effects of cis-9, trans-11 isomer is not clear. The aim of this study was to investigate the effects of CLA with and without Vitamin E on body weight, body composition, glycemic index, inflammatory and coagulation factors, lipid profile, serum leptin and adiponectin, malondialdehyde (MDA), and blood pressure in type2 diabetes.

**Methods:**

56 patients with type2 diabetes were included in 8 week double-blind control trial that used metformin. They randomly divided into three groups: CLA + VitE, CLA + VitE placebo, CLA placebo + VitE placebo. All variables, anthropometric measurements, and body composition were evaluated at the beginning and the end of study. Statistical analysis and analysis of dietary data were performed using SPSS and nutritionist IV software, respectively.

**Results:**

There were not any significant differences in variable changes among three groups. However, there was a trend to increase in MDA and decrease in apoB100 among CLA consumers.

**Conclusion:**

The results of this study showed that administration of CLA supplementation for 8 weeks does not affect any indicators of metabolic control in overweight type2 diabetic patients.

## Background

Conjugated linoleic acid (CLA) is referred to a group of 18 carbon unsaturated fatty acids with two double bond that is isomers of octadecanoic acid [1, 2]. Normally, CLA is produced in the rumen of ruminants through partial biohydration or bacterial fermentation as the first intermediate of linoleic to stearic acid transformation or from trans-11 octadecanoic acid (vaccenic acid) by delta 9-desaturase (oxidation) [3]. Therefore, the major food sources of this unusual fatty acid are ruminant products (meat and milk). Some studies have reported useful properties for CLA such as anti-obesity [4–6], anti-Atherogenic [7, 8], anti-diabetic [9–11] and anti-inflammatory properties [12].

Two active isomers of CLA that have been investigated so far are cis-9, trans-11 and trans-10, cis-12 that first one is more dominant in dairy products [3, 13]. These isomers have several effects in different biological systems and their effects are similar or different sometimes [2]. Results of some studies have shown that trans-10, cis-12 isomers are the active form of CLA reduces body weight and fat accumulation [5]. However, in some cases this isomer lead to worsening peripheral insulin sensitivity and increased concentrations of blood glucose, free fatty acids, and lipid profiles [14–17] and cis-9, trans-11 isomer reduces serum triglyceride levels and plasma free fatty acids [2]. However, the results of 50:50 isomer blend of CLA on weight, body fat, insulin resistance index and inflammation are controversial [10, 18].

Animal studies have shown that isomer trans-10, cis-12 isomers of CLA increase insulin resistance, but the results of studies on cis-9, trans-11 isomer and mixtures (50:50) is contradictory. In some animal studies, CLA had no effect on the blood glucose and insulin resistance [12, 19]. Also, various researches have shown that CLA, even in different species of animals have different effects. For example, in rats, CLA increases PPAR-γ expression as a ligand for this receptor and in this way plays its role in controlling insulin resistance. Also, CLA decreases insulin resistance in adipose tissue of rats. But some studies have shown that CLA increases insulin resistance on mice [19].

Also, the results of CLA supplementation on type II diabetic patients are inconsistent. One of the possible reasons may be the types of consuming drugs, heterogeneity of population in the cases of age, BMI and etc. [7, 20].

In some studies supplementation of CLA led to increment in oxidative stress and inflammation [9, 21]. Since these conditions are related to diabetes, we suppose that supplementation with vitamin E may compensate the possible effects of CLA on oxidative stress and diabetes control. Furthermore, regarding the decrement of Antioxidant capacity in diabetic patients, it raises the possibility that this issue in previous studies may mask some effects of CLA or lead to a slight increase in insulin resistance. Regarding the possible intake of CLA supplements among type 2 diabetic patients to lose weight and tendency of some food industries such as dairy products and oil to enrich their products with CLA,

So, the aim of current study is to investigate the effects of CLA alone and combined with VitE on glycemic response, cardiovascular risk factors, inflammatory indicators, malondialdehyde (MDA) and some adipocytikines associated to diabetes and obesity. Important notable excellence of our study is that to date no well designed controlled study with such inclusion criteria on type 2 diabetic patients has been done.

## Methods

This study was approved by the Ethic Committee of Institute of Food Technology and Nutrition research, Shahid Beheshti University of Medical Sciences and was conducted in 2008 at Research Institute for Endocrine Sciences (RIES), Tehran, Iran.

Sixty-three participants with type2 diabetes whose diabetes was controlled by metformin were recruited and 56 completed the trial. At the beginning, the protocol and the aim of study were fully explained to the participants and written informed consent was obtained from each volunteers. Inclusion criteria, including diagnosed type2 diabetes mellitus for more than 5Y, diagnosis after 30 years old, age of 35 to 50Y, body mass index (BMI; in kg/m^2^) >25 and <30 and fasting blood glucose of 126–180 mg/dl. Exclusion criteria were the history of myocardial infarction, angina pectina and stroke, diagnosis of cardiovascular disease, liver or renal disease or chronic inflammatory and thyroid disease, being vegetarians or vegans, smoking, consuming of alcohol and any supplements (e.g. vitamins such as C and E, fish oils, CLA, etc.) 2 months before intervention, pregnancy and menopause.

The study was an eight weeks randomized double blind, placebo-controlled (RCT) parallel intervention. The participants were stratified according to their sex, age and BMI into one of three groups (A, B, and C) receiving 3.0 g CLA/d (3×1 g capsules; a 50:50 isomer blend of c-9, t-11 and t-10, c-12 CLA) with 100 IU/d VitE, 3.0 g CLA/d with VitE placebo, or CLA placebo (soy bean oil) and VitE placebo respectively. Ineffectiveness of soybean oil as a placebo in the quantities being used in this study on reviewed variables has shown in other studies [20]. The CLA used in this study was Tonalin SG1000T FFA and soft gelatin capsules with clear, transparent shell and pale amber fill. They contain Tonalin FFA 80 i.e., free fatty acids containing about 80% conjugated Linoleic. All supplements were supplied by Cognis, Norway. Each volunteers received the capsules in 2 batches, at the beginning of the study. All participants were asked to maintain their usual physical activity and dietary and lifestyle habits and these were checked by food record and physical activity questionnaire. There was no change in prescribed medication throughout the trial.

Acceptance of the supplements was investigated via weekly phone calling and meetings through asking and counting the remaining capsules in the package delivered. During these calls possible problems such as supplement intolerance and medication use, possibly changes in food consumption, getting a new disease or a change in physical activity was followed and if this situation occurred, the patient desired were excluded. In the fourth and eighth weeks of study through counting the remaining capsules, compliance rates of patients was evaluated and patients were not consumed more than ten percent of received capsules, were excluded from the study.

To evaluate the mean dietary intake two 1-d 24-h food recall and 2-d food records (one day a week holiday) were used on the baseline and the end of the fourth and eighth week of the study. In the first taking the 24 days recall, how to diet properly recording, including how to weigh and measure food was trained by an expert. This dietary information was analyzed with N4 software (Nutritionist: version 4.0; Tinuviel Software, Warrington, United Kingdom).

Body weight was measured with light clothing but no shoes on a digital balance (with 0.1 kg sensitivity). Height was assessed by using a stadiometer that measured to the nearest 0.1 cm. Body mass index (BMI) was estimated as the ratio of body weight to height squared and expressed as kg/m^2^. Waist circumference (with 0.1 cm sensitivity) was measured at the minimum circumference between the iliac crest and the last rib cage at the end of exhalation. The hip circumference was measured using tape as the maximal circumference over the hip and Waist-to-hip ratio (WHR) was calculated [22]. Bioelectrical impedance analysis was used to measurement of body fat and lean percentage (Body Stat 1500, Douglas Isle of man, British islets, England). For this purpose, first two glasses of water consumed by patients and after one hour and urination without any metal on the body, body composition was measured. Measuring body composition was done in lying state and installing the electrodes to the right hand and foot. Patients were taught avoiding intense activity the day before testing [23]. These measurements were done at the beginning and end of the study.

Blood pressure measuring was done to the nearest 2 mmHg, after resting for at least 15min and sitting on the seat handle. Right arm blood pressure was measured, twice, at least five minutes interval, by Korotkoff’s auscultatory method at the beginning and end of the study.

Seven ml 12-hour fasting state and 3 ml postprandial brachial vein blood samples were taken at the baseline and end of eighth week and blood collected into EDAT containing tubes. A standard breakfast in diabetic patients is contained about 360kcal, including: 56.6 g carbohydrate (50-55% Cal), 19.5g protein (15-20% Cal) and 11.5 g fat (30% Cal) [24, 25]. Localized standard breakfast for Iranians that has been used in previous studies included two servings bread; one serving cheese, 2x dates, and four small cubes that total provided about 390kcal and 14g protein [26]. To maintain comparability of Iranian studies with the international studies in which the breakfast induced energy is 360 kcal, and also given that patients participating in this study did not normally use refined s such as in the diet, of breakfast was removed and replaced as a number of dates were added to the breakfast.

Serum glucose concentration was measured by using enzymatic colorimetric method according glucose oxidase principle (Glucose determination kit, Parsazmun, Tehran, Iran) through auto-analyzer instrument (Sellectra II, Dieren, Netherland). Serum insulin, proinsulin and C-peptide levels were measured by Enzyme Linked Immuno assay (ELISA) kit (Mercodia AB, Uppsala, Sweden). Glycated hemoglobin was determined on whole blood sample by ion exchange chromatography method (HbA1c Kit, Inter Medical, Villaricca, Italy).

The intra assay coefficient of variation (CV%) for glucose, insulin, proinsulin, C- peptide and HbA1c were 4.7%, 5.5%, 4.5%, 4.7% and 5.6% and the inter assay coefficient of variation were 4.9%, 5.8%, 4.9%, 5%, and 5.8% respectively. The assays sensitivity was 1mg/dl, 1mU/L, 0.5 pmol/l, 5 pmol/l and 1% respectively.

Insulin resistance was calculated according the homeostasis model of assessment ratio (HOMA-IR) formula, as an index of insulin resistance:

[Insulin (μU/ml)×glucose (mmol/L)]/22.5 [27]. That 5<HOMA IR is defined as insulin resistance and 3> HOMA IR as not-insulin resistance [28].

Insulin sensitivity was calculated from following formula [29]:

$$\mathrm{QUICKI}=\left[1/\left({\log\;\mathrm{glucose}}_{0}+{\log\;\mathrm{insulin}}_{0}\right)\right].$$

And beta cell function determined through proinsulin/ insulin ratio [30]. Beta cell function index as HOMA-B% and C-peptide to insulin molar ratio as an index of liver insulin clearance was calculated [31].

Fasting b-cell responsiveness (M0) represents the ability of fasting glucose to stimulate b-cell secretion and postprandial b-cell responsiveness (M1) represents the ability of postprandial glucose to step up b-cell secretion and they were calculated using the formula of Hovorka et al. [32].

$$M0=100\times \mathrm{fasting}\;C–\mathrm{peptide}\;\left(\mathrm{\mu g}/l\right)/\mathrm{fasting}\;\mathrm{glucose}\;\left(\mathrm{mg}/\mathrm{dl}\right)\;$$

$$\begin{array}{c} M1=100\times (\mathrm{postprandial}\;2h\;C-\mathrm{peptide}-\mathrm{fasting}\;C-\mathrm{peptide}/\\ \;\left(\mathrm{postprandial}\;2h\;\mathrm{glucose}–\mathrm{fasting}\;\mathrm{glucose}\right) \end{array}$$

Serum concentration of triglyceride and HDL cholesterol were measured using kits and enzymatic colorimetric method (Parsazmun, Tehran, Iran). Total cholesterol Concentration was measured by enzymatic photometric method kits (Parsazmun, Tehran, Iran). LDL cholesterol concentration calculated using the Friedewald formula. Also, the ratio of LDL to HDL cholesterol concentrations was calculated. The intra assay coefficient of variation (CV%) for triglycerides, total cholesterol and HDL cholesterol were 2.7%, 2.3% and 5.1% and the inter assay coefficient of variation were 2.9%, 2.5T and 5.5% respectively. Also, the related assays sensitivity was 1 mg/dL, 3mg/dL and 1mg/dl respectively.

Serum leptin levels were measured by Enzyme Linked Immuno assay (ELISA) kit (Diagnostics Biochem Canada Inc., ontario, Canada). Serum adiponectin levels were measured by Enzyme Linked Immuno assay (ELISA) kit (Mercodia AB, Uppsala, Sweden). The intra assay coefficient of variation (CV%) for leptin and adiponectin were 2% and 1.5% respectively and the inter assay coefficient of variation were 4% and 2%. The sensitivity of the assays was 0.50 ng/ml and 1.25 ng/ml respectively.

Serum concentrations of interleukin-1 beta, interleukin-6 and TNF-α were measured using ELISA kits (Diaclone, France) and CRP concentration using ELISA kit (Diagnostics Biochem Canada Inc., Ontario, Canada). Intra assay coefficient of variation of inflammatory IL-1 beta, interleukin 6, CRP and TNF-α, were 7.1%, 4.6%, 1.8%, 3% and the related inter assay coefficient of variation were 7%, 4.7%, 2% and 3.2% respectively. Sensitivity of assays were also 7 pg/ml, 2 pg/ml, 10 ng/ml and 8 pg/ml. Measuring fibrinogen concentration and PAI-1was done using ELISA kits (Hyphen BioMed, Neuville-Sur-Oise, France) and MDA concentration by using the colorimetric method kit (Cayman Chemical Company, Ann Arbor, USA). The intra and inter assay coefficient of variation for fibrinogen and PAI-1, were 2.6%, 3.2% and 3%, 3.5% respectively and assays sensitivity were 1 mg/ml and 0.5 ng/ml respectively. These values for apoB-100 and MDA were 1.4%, 3% and 2%, 3.5% and 0.1 μg/dl, 1 μmol/L respectively.

### Statistical analysis

All statistical tests were performed with the use of SPSS (version 13.0; SPSS Inc, Chicago) and a p-value<0.05 showed statistical significance. Normality and homogeneity of variance was tested with Kolmogrov-Smirinov. ANOVA test were used to compare mean differences before and after intervention for anthropometric data and body composition. Other changes were compared using ANCOVA test adjusted to body composition and waist circumference. To compare food consumption at the baseline and end of the fourth and eighth weeks among groups, and comparison of food consumption in each group among baseline and end of the fourth and eighth weeks of study the ANOVA test and repeated measure ANOVA test were used.

## Results

Among 63 individuals participated in this research, one patient was excluded due to reporting heartburn and digestive discomfort, one due to herbal drug use and five patients due to not taking the supplements regularly. At the end, 56 (30females and 26males) completed the study. Baseline characteristics of patients of A, B, and C groups listed in Table 1.

**Table 1:** **Baseline characteristics of patients**

	A group (mean ± SD)	B group (mean ± SD)	C group (mean ± SD)
Age (year)	47.6 ± 3.8	45.1 ± 5.7	45.5 ± 4.3
Sex (N)			
Male	8	9	9
Female	9	10	11
Weight (Kg)	77.2 ± 13.1	77.6 ± 10.9	73.1 ± 9.4
BMI (Kg/m^2^)	28.1 ± 2.4	27.4 ± 0.5	27.1 ± 1.8
Waist circumference (cm)			
Male	96.4 ± 9.2	93.0 ± 7.2	91.1 ± 5.7
Female	86.3 ± 9.2	88.8 ± 7.7	89.0 ± 5.8
Fat %			
Male	28.1 ± 3.8	22.2 ± 7.8	26.1 ± 7.2
Female	41.5 ± 6.0	40.5 ± 7.8	34.1 ± 8.0
FBG (mg/dl)	155.5 ± 15.4	154.1 ± 14.1	155.5 ± 20.2
HbA1c%	9.4 ± 1.6	10.4 ± 1.5	10.4 ± 1.7

*BMI* Body Mass Index, *FBG* Fasting Blood Glucose.

A group: 3.0 g CLA/d (3×1 g capsules; a 50:50 isomer blend of c-9, t-11 and t-10, c-12 CLA) with 100IU/d VitE.

B group: 3.0 g CLA/d with VitE placebo.

C group: CLA placebo (soy bean oil) and VitE placebo.

Patients’food intakes at the baseline, the end of the fourth and eighth weeks has been shown in Table 2. During the study, participants’ food intake did not change significantly. It is notable that we analyzed dietary SFA, MUFA and PUFA and also, physical activity level and no changes were shown. Data was not shown in article.

**Table 2:** **Dietary intake in patients with type 2 diabetes** (**experimental group**=**17**, = **21 positive controls and negative controls** = **20**)

Food groups and nutrients	Beginning (mean ± SD)	4 wk (mean ± SD)	8 wk (mean ± SD)	p-value
Dairy (servings)				
A (N=17)	2.0 ± 0.3	2.2 ± 0.28	2.7 ± 0.27	0.95
B (N=21)	1.9 ± 0.25	2.2 ± 0.23	2.0 ± 0.15	0.95
C (N=20)	1.8 ± 0.12	1.7 ± 0.25	1.9 ± 0.23	0.93
Meat (serving)				
A (N=17)	2.1 ± 0.23	2.0 ± 0.35	2.2 ± 0.23	0.8
B (N=21)	1.9 ± 0.30	1.9 ± 0.25	1.8 ± 0.27	0.9
C (N=20)	2.0 ± 0.21	1.8 ± 0.23	1.8 ± 0.11	0.8
Cereals (serving)				
A (N=17)	11.12 ± 2.03	12.75 ± 1.76	11.5 ± 2.55	0.85
B (N=21)	12.15 ± 1.05	12.75 ± 0.3	12.12 ± 1.12	0.8
C (N=20)	11.43 ± 1.11	12.34 ± 0.81	12.23 ± 0.91	0.89
Fruit (serving)				
A (N=17)	2.2 ± 0.09	2.65 ± 0.49	2.0 ± 0.54	0.82
B (N=21)	1.9 ± 1.01	2.09 ± 0.45	2.0 ± 0.87	0.8
C (N=20)	1.8 ± 1.12	1.8 ± 1.54	2.01 ± 1.12	0.9
Vegetables (serving)				
A (N=17)	1.6 ± 0.27	1.9 ± 0.14	1.7 ± 0.34	0.79
B (N=21)	1.3 ± 0.09	2.1 ± 0.65	1.4 ± 0.21	0.88
C (N=20)	1.4 ± 0.11	1.6 ± 0.23	1.6 ± 0.65	0.65
Energy (Kcal)				
A (N=17)	1996 ± 198	1919 ± 255	1983 ± 211	0.82
B (N=21)	1978 ± 234	1950 ± 199	1975 ± 240	0.7
C (N=20)	1999 ± 222	1980 ± 198	2003 ± 201	0.76

Table 3, 4, and 5 summarize the results of supplementation on the studied variables.

**Table 3:** **Mean and standard deviation of the anthropometric measurements and body composition in 3 groups**

	Time = 0			Time = 8 wk			
	Group A (mean ± SD)	Group B (mean ± SD)	Group C (mean ± SD)	Group A (mean ± SD)	Group B (mean ± SD)	Group C (mean ± SD)	p^α^
Weight (Kg)	77.2 ± 13.1	77.6 ± 10.9	73.1 ± 9.4	76.9 ± 12.8	77.0 ± 11.1	72.7 ± 9.5	0.86
BMI (Kg/m^2^)	28.1 ± 2.4	27.4 ± 0.5	27.1 ± 1.8	28.0 ± 2.7	27.2 ± 2.7	26.9 ± 1.8	0.91
WC (cm)							
Female	86.3 9.2	88.8 ± 7.7	89.0 ± 5.8	84.0 ± 8.3	85.5 ± 10.2	88.0 ± 5.8	0.41
Male	96.4 ± 9.8	93.0 ± 7.2	91.1 ± 5.7	96.2 ± 8.1	91.1 ± 5.7	92.0 ± 5.7	0.51
WHR							
Female	0.84 ± 0.08	0.83 ± 0.04	0.90 ± 0.05	0.83 ± 0.07	0.82 ± 0.05	0.89 ± 0.06	0.84
Male	0.95 ± 0.04	0.91 ± 0.03	0.95 ± 0.06	0.96 ± 0.05	0.91 ± 0.03	0.94 ± 0.05	0.28
Fat%							
Female	41.5 ± 6.0	40.5 ± 7.8	34.1 ± 8.0	39.3 ± 6.2	38.2 ± 6.3	38.7 ± 7.6	0.24
Male	28.1 ± 3.8	22.2 ± 7.8	26.1 ± 7.2	26.7 ± 7.0	26.8 ± 10.8	27.1 ± 6.4	0.12
Lean%							
Female	58.8 ± 5.7	59.5 ± 7.8	65.8 ± 8.0	60.6 ± 6.2	61.6 ± 6.3	61.0 ± 7.6	0.25
Male	71.8 ± 3.8	77.7 ± 7.8	73.8 ± 7.2	73.2 ± 7.0	73.1 ± 10.8	72.8 ± 6.4	0.12

*BMI* Body Mass Index, *WC* Waist circumference, *WHR* Waist to Hip Ratio, *Fat%* Percent of Body Fat Mass, *Lean%* Percent of Lean Body Mass.

α: P values are related to the comparison of variables change differences at the beginning and end of the study between three groups.

**Table 4:** **Mean and standard deviation of fasting and postprandial glycemic indicators**, **lipid profile**, **apo B**_**100**_, **MDA and blood pressure at the beginning and end of study**

	Time = 0			Time = 8 wk			
	Group A (mean ± SD)	Group B (mean ± SD)	Group C (mean ± SD)	Group A (mean ± SD)	Group B (mean ± SD)	Group C (mean ± SD)	p^α^
FBG (mg/dl)	155.5 ± 15.4	154.1 ± 14.1	155.5 ± 20.2	151.3 ± 17.9	156.8 ± 20.6	153.3 ± 19.1	0.52
Fasting insulin (mIU/L)	8.7 ± 6.8	5.1 ± 3.2	10.1 ± 9.6	8.4 ± 6.2	5.6 ± 3.4	10.9 ± 10.2	0.83
Fasting proinsulin (pmol/L)	19.7 ± 15.0	13.1 ± 9.1	19.7 ± 18.2	18.7 ± 18.1	10.9 ± 7.3	17.1 ± 14.1	0.36
Fasting C peptide (pmol/L)	701.9 ± 272.0	690.3 ± 356.1	604.4 ± 235.3	672.7 ± 319.1	570.7 ± 259.4	582.6 ± 300.3	0.66
QUICKI	0.328 ± 0.023	0.343 ± 0.040	0.320 ± 0.052	0.334 ± 0.028	0.353 ± 0.040	0.338 ± 0.052	0.81
HOMA-IR	3.34 ± 2.76	2.84 ± 2.50	8.88 ± 19.13	3.07 ± 1.18	2.14 ± 1.18	4.12 ± 4.02	0.54
Fasting proinsulin/insulin	2.73 ± 2.33	2.79 ± 2.54	3.01 ± 2.82	3.32 ± 3.57	2.51 ± 2.25	3.24 ± 3.29	0.53
β cell responsiveness	1.29 ± 0.45	1.37 ± 0.72	1.20 ± 0.54	1.31 ± 0.68	1.16 ± 0.68	1.00 ± 0.65	0.54
HOMA B%	34.56 ± 25.26	30.81 ± 25.19	52.83 ± 76.04	36.36 ± 27.36	24.81 ± 17.81	46.74 ± 46.18	0.44
2h-BS(mg/dl)	191.0 ± 18.6	177.1 ± 21.4	187.4 ± 20.1	174.3 ± 24.8	184.2 ± 28.5	191.2 ± 29.3	0.18
2h- insulin(mIU/L)	20.6 ± 12.3	21.1 ± 12.1	26.3 ± 21.1	14.7 ± 5.7	22.1 ± 12.3	20.1 ± 17.4	0.49
2h-proinsulin(pmol/L)	36.4 ± 22.1	25.3 ± 11.2	38.1 ± 28.2	31.8 ± 28.3	26.5 ± 15.3	36.3 ± 19.6	0.77
2h-C peptide(pmol/L)	1490.4 ± 579.6	1466.1 ± 600.9	1432.4 ± 613.1	1316.1 ± 379.7	1457.1 ± 705.7	1324.1 ± 363.2	0.29
2h- proinsulin/insulin	1.94 ± 1.18	1.58 ± 1.31	2.28 ± 1.77	2.40 ± 2.00	1.49 ± 1.11	2.30 ± 1.18	0.3
HbA1c%	9.43 ± 1.64	10.47 ± 1.59	10.40 ± 1.78	8.93 ± 1.12	9.46 ± 1.70	9.33 ± 1.54	0.68
TG (mg/dl)	185.9 ± 58.9	193.1 ± 78.7	227.2 ± 81.1	172.2 ± 58.8	165.2 ± 58.5	182.8 ± 58.9	0.19
TC (mg/dl)	198.8 ± 27.3	223.5 ± 43.1	234.1 ± 44.3	194.8 ± 37.6	223.2 ± 55.8	227.2 ± 37.2	0.81
LDL-C (mg/dl)	112.9 ± 25.5	132.2 ± 28.5	135.2 ± 40.5	118.8 ± 24.5	144.2 ± 45.9	142.4 ± 33.1	0.97
HDL-C (mg/dl)							
Female	47.6 ± 6.1	46.1 ± 5.1	50.6 ± 9.2	40.4 ± 6.2	40.3 ± 8.3	51.3 ± 6.6	0.97
Male	48.1 ± 4.7	56.1 ± 10.1	51.4 ± 12.2	42.8 ± 5.7	48.9 ± 9.1	43.8 ± 4.1	0.6
LDL/HDL							
Female	2.5 ± 0.5	2.7 ± 0.5	3.1 ± 1.2	2.9 ± 0.2	3.8 ± 1.6	2.9 ± 0.6	0.22
Male	2.2 ± 0.4	2.4 ± 0.4	2.4 ± 0.8	2.7 ± 0.4	2.9 ± 0.5	3.0 ± 0.6	0.97
Apo B_100_ (mcg/dl)	61.8 ± 12.4	68.0 ± 13.1	62.6 ± 11.9	53.6 ± 13.0	58.8 ± 14.8	62.7 ± 14.2	0.84
MDA (mcmol/L)	14.2 ± 6.0	12.1 ± 5.1	11.6 ± 6.7	16.1 ± 8.1	16.5 ± 8.0	11.2 ± 6.7	0.62
Systolic blood pressure (mmHg)	126.5± 12.8	115.4±10.1	130.1± 23.1	125± 13.5	115.2± 9.3	128.3± 19.1	0.81
Diastolic blood pressure(mmHg)	87.6± 8.3	78.1± 7.3	84.4± 11.1	83.4± 12.4	79.1± 7.2	84.3± 9.2	0.21

*FBG* Fasting Blood Glucose , *QUICKI* Insulin sensitivity Index, *HOMA-IR* Homeostasis Model of Assessment Insulin Resistance, *HOMA B%* Beta cell function index, *HbA1c%* percent of glycosilated hemoglobin, *2h-BS* 2 Hour postprandial Blood Glucose, *TG* Triglyceride, *TC* Total Cholesterol, *LDL-C* low Density Lipoprotein Cholesterol, *HDL-C* High Density Lipoprotein Cholesterol, *Apo B*_*100*_ Apoprotein B_100_, *MDA* Malondialdehyde.

Group A- receiving 3.0 g CLA/d (3×1 g capsules; a 50:50 isomer blend of *c*-9, *t*-11 and *t*-10, *c*-12 CLA) with 100 IU/d vitamin E, Group B- receiving 3.0 g CLA/d with vitamin E placebo, Group C- receiving CLA placebo and vitamin E placebo.

α: P values are related to the comparison of variables change differences at the beginning and end of the study between three groups by ANCOVA test.

**Table 5:** **Mean and standard deviation of inflammatory indicators**, **leptin**, **adiponectin**, **Fibrinogen and PAI**-**1 at the beginning and end of study**

	Time = 0			Time = 8 wk			
	Group A (mean ± SD)	Group B (mean ± SD)	Group C (mean ± SD)	Group A (mean ± SD)	Group B (mean ± SD)	Group C (mean ± SD)	p^α^
IL-1β (pg/ml)	17.6 ± 10.9	20.2 ± 15.1	14.3 ± 7.2	13.5 ± 7.2	13.5 ± 7.4	10.7 ± 2.8	0.17
IL-6 (pg/ml)	4.1 ± 2.8	4.1 ± 1.4	4.4 ± 2.1	3.5 ± 1.5	3.2 ± 0.6	4.3 ± 3.2	0.14
CRP (ng/ml)	2138.2 ± 3469.2	2083.0 ± 2362.5	1922.9 ± 2482.5	1159.8 ± 1022.7	1533.8 ± 1189.8	1564.0 ± 1943.3	0.38
TNF-α (pg/ml)	15.1 ± 2.9	14.9 ± 2.5	14.4 ± 3.5	12.3 ± 2.9	11.9 ± 2.0	11.9 ± 1.9	0.74
Leptin (ng/ml)							
Female	23.40 ± 12.42	23.44 ± 11.57	10.74 ± 4.79	20.13 ± 14.91	14.55 ± 6.10	6.61 ± 0.98	0.54
Male	6.47 ± 5.66	9.32 ± 7.66	4.90 ± 2.60	6.92 ± 6.48	5.86 ± 5.47	2.98 ± 1.57	0.11
Adiponectin (ng/ml)	61.43 ± 19.96	76.0 ± 26.82	62.38 ± 26.74	62.06 ± 21.24	71.89 ± 29.10	61.90 ± 19.96	0.55
Fibrinogen (mg/dl)	67.0 ± 6.4	69.8 ± 7.1	68.9 ± 7.1	63.1 ± 8.7	64.4 ± 7.8	61.3 ± 9.3	0.56
PAI-1 (ng/ml)	18.5 ± 16.2	17.3 ± 11.2	14.8 ± 6.0	18.0 ± 16.6	20.7 ± 18.1	15.0 ± 11.6	0.73

*IL-1β* interleukin 1 beta, *IL-6* interleukin 6, *CRP* C Reactive Protein, *TNF-α* Tumor Necrosis Factor, *PAI-1* Plasminogen activator inhibitor-1.

Group A- receiving 3.0 g CLA/d (3×1 g capsules; a 50:50 isomer blend of *c*-9, *t*-11 and *t*-10, *c*-12 CLA) with 100 IU/d vitamin E, Group B- receiving 3.0 g CLA/d with vitamin E placebo, Group C- receiving CLA placebo and vitamin E placebo.

α: P values are related to the comparison of variables change differences at the beginning and end of the study between three groups by ANCOVA test.

## Discussion

In this study, consumption of CLA alone and in combination with VitE did not result in significant changes in any of the measured variables.

So far, few studies regarding the effect of CLA has been done in diabetic patients. In a study, usual dose of commercial CLA supplement improved the insulin sensitivity index in young diabetic adults, but some differences were observed in their responsiveness [10]. In another study, supplemental CLA significantly increased fasting glucose concentrations and reduced insulin sensitivity (homeostasis model), oral glucose insulin sensitivity and insulin sensitivity index (ISI) [7], while in our study any of related parameters were not affected. Inconsistency in the results of studies on diabetic patients may be due to differences in patients ‘responsiveness. As shown in Eyjolfson et al. study, despite average increment in insulin sensitivity with 4g supplementation, two participants demonstrated essential no, and two had a decrease in insulin sensitivity. In that study, among six patients, insulin sensitivity found to increase and therefore caused to increase in the average insulin sensitivity in the intervention group [23]. Elusive changes in blood biochemistry presented in our study may also be due to differences in the patients’ responsiveness. This could be caused by differences in duration of diabetes, patients ‘weight, the severity of insulin resistance and other parameters measured at baseline and different amounts of CLA intake from the usual diet. But what is clear, in patients with normal BMI or overweight, CLA has no or a little effect on improvement of blood glucose, insulin and insulin sensitivity [10, 33]. So, it is likely that in obese individuals, CLA causes a small increase in insulin resistance [9, 34]. However in our study, CLA supplementation did not change weight and glycemic indicators.

In most studies, serum glucose, insulin, and sometimes, fasting pro insulin and C-peptide have been measured and only indicators of insulin resistance and insulin sensitivity has been reported. While, several studies have shown that serum pro insulin, pro insulin/insulin are better predictors of the risk of heart disease compared to insulin concentration [35]. In T2D pro insulin, insulin ratio increases, that can be the result of insulin resistance or impaired conversion of pro insulin to insulin [36]. In the present study, serum concentrations of C peptide and pro insulin were also measured at the beginning and end of the study and no changes were detected.

Recently, using pure isomers of CLA has shown that the effects of CLA on body composition that is related to trans-10, cis-12 isomer [6, 37]. This fatty acid decreases appetite and energy intake in laboratory animals, increases energy expenditure in the body by increasing expression of Mitochondrial uncoupling protein 2 (UCP2). CLA decreases lypogenesis through inhibition of lipoprotein lipase (LPL2), fatty acid synthase, acylcoenzyme A carboxylase and PPAR-γ. Also, CLA increases fat oxidation. Thus, CLA reduces fatty cell size with no effect on cell numbers [5]. Changes in weight and body composition observed in human studies are not as changes observed in experimental animals. In some human studies, CLA supplementation was associated to decrease in body weight and fat mass [21, 38], but in others there was not any changes in body composition [39, 40]. It seems that factors such as current state of health, degree of obesity, and activity levels might influence the impact that CLA supplementation has on altering body composition. Also, given the fact that the molecular mechanism of CLA is through changing gene expression [12, 39], the differences in gene expression in several species can cause contradictory results. Regarding human studies, Belury and colleagues showed that plasma levels of CLA has been inversely associated with body weight and the trans-10, cis-12 isomer is responsible for this negative correlation and biological active responsible for weight change in people with type2 diabetes. Short duration of intervention in these studies may be one reason of elusive changes in anthropometric measurements, body fat and lean body mass. In the present study the daily intake of 3g of CLA capsules for eight weeks, did not occur significant changes in weight, BMI, waist circumference and body fat mass percentage. However, the results of this study is not comparable to the results of separated isomers, because the commercial CLA supplements contain the two active isomer in equal ratio, so each isomers differentially act on expression of related enzymes [12]. In long-term studies of 6 and 12 months using supplemental CLA isomers in equal ratio, weight loss and a reduction in body fat mass have been reported [41, 42]. It seems that appearing of CLA effects on weight and body composition, more time is needed. As in our study which lasted eight weeks, no changes in physical parameters were seen. However, in some three months and even six-week studies changes in body weight and fat mass have been seen [42, 43]. The differences in individuals’ response in expression of enzymes associated with energy expenditure and body composition, type of drugs and CLA dietary intake can justify the inconsistency in the results of studies. As this study was on glucose-lowering drug consumers that may interfere with the mechanisms of CLA in some metabolic pathways. Also, calculation of dietary CLA intake was not possible.

In this study, daily supplementation with 3 g of CLA capsules alone and combined with 100 IU of VitE for eight weeks compared to placebo, did not result in significant changes in serum triglycerides, total cholesterol, LDL, HDL cholesterol and apoB100. Some animal studies using pure CLA isomers have shown that cis-9, trans-11 isomer lead to lowering triglyceride levels and plasma free fatty acids and trans-10, cis-12 isomer lead to increasing plasma free fatty acids and LDL cholesterol [2] that is confirmed by human studies [21]. However, the results of equal ratio of isomers are contradictory. In a study on patients with type2 diabetes, taking 3g of CLA supplementation for 8 weeks, resulting in increased HDL_2_ cholesterol and decreased LDL to HDL cholesterol ratio [7], although in another study, reduced serum HDL cholesterol following 12 weeks of supplementation has been reported [44]. In other studies with 6 weeks and 6 months duration, no significant changes were shown in serum lipids and Apo proteins [33, 43]. Overall, the effects of commercial CLA capsules on lipids profiles are unknown. Further studies should be done in this regard to identify the mechanisms of CLA in regulating gene expression involved in lipid metabolism.

In our study, it was not shown any change in systolic and diastolic blood pressure. In two animal studies, CLA has reduced blood pressure [45, 46]. In another study, trans-10, cis-12 isomer lowered blood pressure and cis-9, trans-11 did not affect [47]. CLA effects on blood pressure could be related to the pathway of eicosanoids production. Inhibition of Arashidonic acid released from cell membranes and cyclooxygenase by CLA has been reported [11].

In our study, 3 g daily CLA capsules alone and combined with 100 IU of VitE for eight weeks did not affect any inflammatory parameters IL-1 beta, interleukin-6, TNF-α, CRP, Serum leptin and adiponectin. Animal studies show that trans-10, cis-12 isomer increases indicators of inflammation and cis-9; trans-11 is associated with reduction of inflammatory indicators [12]. In this way the CLA supplementation in rats leads to decreased TNF-α and in mice increased it [19]. In some human studies, CLA did not affect serum inflammatory parameters [7], but in the some others led to increase in these mediators [45], in the present study also no significant changes developed.

In one study, CLA supplementation reduced leptin and adiponectin [8] and in two other studies any changes are not mentioned [9, 28]. In the present study changes in serum leptin and adiponectin showed no statistically differences among the three groups.

In our study there was not seen any significant differences in serum fibrinogen and PAI-1 changes among three groups. Also, no information is regarding the effects of CLA supplementation on these variables.

In the present study 3 g of CLA capsules alone and combined with 100IU VitE did not make a difference in the oxidative stress index, MDA compared with placebo. In some studies, taking an active CLA isomer has increased lipid peroxidation indexes, including urinary excretion of isoprostaglandins [9, 21].

One limitation of this study is that estimation of dietary and serum CLA was not possible. Differences in dietary intake and thus serum CLA levels can affect patients’ responsiveness to supplementation. Also, the use of 2-d diet records may not accurately reflect changes in calories during intervention. Duration of this study was 8 weeks. It may be required longer time to appearance CLA effects.

Because of insufficient information regarding the molecular mechanisms of CLA in humans, human cell culture studies are suggested.

## Conclusion

Overall, our results show that commercial CLA supplementation alone or combined with VitE for 8weeks, does not significantly affect glycemic indicators, body composition, lipid concentrations, indicators of inflammation, coagulation, leptin, adiponectin, MDA and blood pressure in type2 diabetes, but there was a significant trend to increase in MDA and decrease in apoB100 among CLA consumers.

## Abbreviations

ANCOVA: Analysis of covariance
ANOVA: Analysis of variance
BMI: Body mass index
Cal: Calorie
CLA: Conjugated linoleic acid
CRP: C - reactive protein
CV: Coefficient of variation
EDTA: Ethylenediaminetetraacetic Acid
ELISA: Enzyme Linked Immuno assay
FFA: Free fatty acid
HbA1c: Hemoglobin A1C
HDL: High density lipoprotein
HOMA-IR: Homeostasis model of assessment ratio- insulin resistance
ISI: Insulin sensitivity index
LDL: Low density lipoprotein
LPL: Lipoprotein lipase
MDA: Malondialdehyde
PAI-1: Plasminogen activator inhibitor-1
PPAR: Peroxisome proliferator-activated receptor
QUICKI: Quantitative insulin sensitivity check index
RCT: Randomized double blind, placebo-controlled trial
RIES: Research Institute for Endocrine Sciences body mass index
T2D: Type 2 diabetes
TNF-α: Tumor necrosis factor-alpha
UCP2: Mitochondrial uncoupling protein 2
WHR: Waist-to-hip ratio.
